# Biofilm-producing and carbapenems-resistant *Escherichia coli* nosocomial uropathogens: a cross-sectional study

**DOI:** 10.1007/s10123-024-00495-w

**Published:** 2024-03-15

**Authors:** Doaa Abo-alella, Wessam Abdelmoniem, Enas Tantawy, Ahmed Asaad

**Affiliations:** Department of Medical Microbiology and Immunology, Faculty of Medicine, Zagazig University, Zagazig, Egypt

**Keywords:** Uropathogens, *E. coli*, Biofilm, Carbapenem resistance, UPEC

## Abstract

**Objectives:**

This cross-sectional study aims to determine the incidence and potential risk factors associated with biofilm-producing uropathogenic *Escherichia coli* (UPEC) nosocomial strains from a tertiary care hospital and to examine the prospective correlation between biofilm generation and antibiotic resistance phenotypes and genotypes.

**Methods:**

A total of 130 UPEC nosocomial isolates were identified, their biofilm formation was quantified using a modified microtiter plate assay, and their antibiotic susceptibilities were assessed utilizing the disc diffusion method. Isolates were then subjected to PCR assays targeting *bla*_KPC_, *bla*_VIM_, *bla*_IMP_, and *bla*OXA48 genes.

**Results:**

Over half of the isolates (*n* = 76, 58.5%) were biofilm producers. Among 17 carbapenem-resistant isolates, 6 (42.9%) isolates harbored the *bla*_OXA48_ gene, and only 1 (9.1%) isolate was positive for the *bla*_VIM_ gene. Prior antibiotic therapy (aOR 15.782, *p* 0.000) and diabetes mellitus DM (aOR 11.222, *p* 0.016) were the significant risk factors associated with biofilm production, as determined by logistic regression analysis of the data. In addition, gentamicin resistance was the only statistically significant antibiotic resistance pattern associated with biofilm production (aOR 9.113, *p* 0.02).

**Conclusions:**

The findings of this study emphasize the significance of implementing proper infection control measures to avoid the horizontal spread of biofilm formation and associated antimicrobial resistance patterns among UPEC nosocomial strains.

## Introduction

Uropathogenic *Escherichia coli* (UPEC) is considered the most common uropathogens associated with urinary tract infections (UTIs), involving pyelonephritis, cystitis, and infectious complications that can lead to acute renal failure in both healthy individuals and renal transplant recipients (Zagaglia et al. 2022).

The capacity to generate biofilms is essential for the pathogenic role and virulence of UPEC to penetrate, proliferate, ascend, and survive within the uroepithelium (Katongole et al. 2020). Biofilms provide bacteria with a survival strategy by positioning them to use available resources efficiently and limiting their access to antimicrobial drugs, antibodies, and white blood cells (Öztürk et al. 2023). Furthermore, UPEC isolates harbor numerous antibiotic-inactivating enzymes, including ꞵ-lactamases and aminoglycosides transferases, generating an antimicrobial resistance island (Davies and Davies 2010).

This issue is exacerbated by the emergence and spread of multi-drug-resistant (MDR) bacterial strains due to diverse resistance mechanisms, severely limiting the therapeutic options available to clinicians and representing the most significant challenges associated with treating UTIs (Naber et al. 2020). Among UPEC strains harboring carbapenem-resistance genes, the prevalence of MDR is increasing, posing a severe, severe threat to global health (Katongole et al. 2020).

In terms of carbapenem hydrolysis and geographical distribution, the most effective carbapenemases are Verona integron-encoded metallo-beta lactamases (VIM)-type (representing class B β-lactamases) and oxacillin-hydrolyzing carbapenemases (OXA48)-type (representing class D β-lactamases) (Tanriverdi Cayci et al. 2021). Carbapenemase genes are chromosomally or plasmid-encoded on transmissible genetic cassettes inserted into integrons and/or associated with composite transposons, facilitating their intra- and inter-species horizontal dissemination (Reyes et al. 2020). The development of molecular approaches for genetic identification of carbapenem-resistant UPEC has progressed into a sensitive, accurate, and rapid detection technique (Codjoe and Donkor 2017). This study examines the frequency and associated risk factors of biofilm-producing UPEC nosocomial strains from a tertiary care hospital while testing the hypothesis that the presence of biofilm producers increases the risk of antimicrobial resistance (AMR) and MDR.

## Materials and methods

### Study design and ethical approval

This cross-sectional observational study was performed between January 2022 and March 2023 at Zagazig University Hospitals. All study procedures followed the ethical code of the World Medical Association (Declaration of Helsinki) for experiments, including human cases. The Institutional Review Board (IRB) Committee of the Faculty of Medicine, Zagazig University, approved the study protocol (approval No. 63643/29–9-2020). Informed written consent was collected from all subjects who participated in the study. Additionally, the study was conducted in accordance with the international guidelines of Strengthening the Reporting for Observational Studies in Epidemiology (STROBE) (Vandenbroucke et al. 2007).

### Urine sample collection and isolation of UPEC strains

During the study period, non-duplicate UPEC nosocomial strains were collected from adult patients hospitalized for 48 h and classified according to the criteria of the Centers for Disease Control and Prevention/National Healthcare Safety Network (CDC/NHSN) (Horan et al. 2008). Demographic and clinical data of the hospitalized cases were collected utilizing a questionnaire involving age, ward/ICU admission, sex, catheterization, exitance of comorbid conditions, hospital stay's length, and a history of prior antibiotic treatment. In order to isolate UPEC, urine samples were initially screened using nutrient agar, blood agar, MacConkey agar, CLED plates, and the API 20E (bioMerieux, Mar-cy-l’Etoile, France) according to the manufacturer’s instructions (Murray et al. 2020).

### Quantitative tissue culture plate method to assess biofilm formation by UPEC

**Fig. 1:**
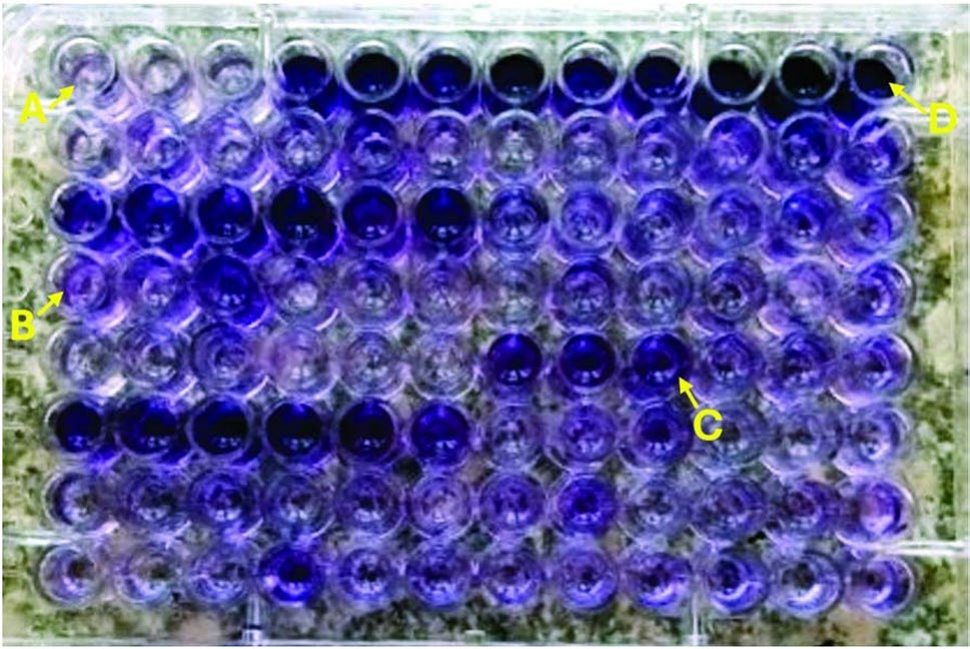
Quantitative tissue culture plate method to assess biofilm formation by *E. coli* uropathoegnic isolates after crystal violet staining. (A) Negative control, (B) weak biofilm formation, (C) moderate biofilm formation, and (D) strong biofilm formation

The production of biofilm by UPEC was evaluated phenotypically using the quantitative tissue culture plate method (Hassan et al. 2011). Isolates from fresh agar plates were inoculated into 10 mL of trypticase soy broth with 1% glucose. Culture turbidity was adjusted to a 0.5 McFarland standard. Individual wells of sterile 96-well flat-bottom polystyrene tissue culture plates (Costar, CA, USA) were added with 200 µL of the bacterial suspension. Negative control wells contained sterile broth. The reference strain *E. coli* ATCC 25922 was utilized as the positive control. Plates were incubated at 37 °C for 24 h. After incubation, the content of each well was gently removed. The wells were washed three times with 200 µL of phosphate buffered saline (pH 7.2) to remove free-floating planktonic bacteria, and then the wells were air dried for 45 min. Adherence of bacteria to the culture plate was detected using crystal violet by adding 200 µL of 0.1% crystal violet to each well, and the plates were incubated at room temperature for 10 min. The excess stain was removed by rinsing with deionized water, and the plates were allowed to dry for 20 min. After drying, 200 μL of 95% ethanol was added to the wells to solubilize the incorporated dye; the plate was covered with the lid (to minimize evaporation) and left at room temperature for 30 min. The ODs of the stained adherent bacteria were measured with a microplate reader at 570 nm. Experiments were performed in triplicate (Fig. 1). The average OD values were calculated for all tested isolates and negative controls, and the cutoff value (ODc) was detected. It is defined as three standard deviations (SDs) above the mean OD of the negative control. The final OD value of a tested strain was expressed as the average OD value of the strain reduced by the ODc value. Negative values were presented as zero, whereas positive values indicated the formation of biofilm. Strains were divided into the following categories to facilitate interpretation of the results: non-biofilm producer = OD ≤ ODc; weak biofilm producer = ODc < OD ≤ 2 × ODc; moderate biofilm producer = 2 × ODc < OD ≤ 4 × ODc, and strong biofilm producer = 4 × ODc < OD (Stepanović et al. 2007; Panda et al. 2016).

### Antibiotic susceptibility testing

Antibiotic susceptibility patterns were detected using the disc diffusion method on Muller-Hinton agar plates according to CLSI guidelines (CLSI 2022). The antibiotics tested included nitrofurantoin (NIT, 300 µg), doxycycline (DOX, 5 µg), tigecycline (TGC, 15 µg), trimethoprim/sulfamethoxazole (SXT, 1:19, 25 µg), levofloxacin (LVX, 30 µg), ciprofloxacin (CIP, 5 µg), amikacin (AMK, 30 µg), gentamycin (GEN, 10 µg), meropenem (MEM, 10 µg), imipenem (IPM, 10 µg), aztreonam (ATM, 30 µg), cefoxitin (FOX, 10 µg), ceftazidime (CAZ, 30 µg), cefotaxime (CTX, 30 µg), cefepime (FEP, 30 µg), piperacillin/tazobactam (TPZ, 100/10 µg), ampicillin/sulbactam (SAM, 10/10 µg), and amoxicillin/clavulanic acid (AMC, 20/10 µg) (Oxoid, England). The reference strain *E. coli* ATCC 25922 was utilized for quality control.

### Multiplex PCR assays to detect *bla*KPC, *bla*IMP, *bla*VIM, and *bla*OXA48 genes

All carbapenem-resistant isolates were subjected to multiplex PCR to detect *bla*_KPC_, *bla*_IMP_, *bla*_VIM_, and *bla*_OXA48_ genes, as previously described (Baran and Aksu 2016). The primers utilized in this study and the amplicon sizes are listed in Table 1. PCR was performed in two multiplex reactions of a total volume of 20 µL for each run. The first reaction for detecting *bla*_KPC_ and *bla*_OXA48_ contained 10 µL of 2X PCR Master mix solution (DreamTaq Green PCR Master Mix, Thermofisher Scientific, USA), 1 µL of each primer, 4 µL of template DNA, and sterile distilled water to a total volume of 20 µL. The second reaction for detecting *bla*_IMP_ and *bla*_VIM_ contained 10 µL 0f 2X PCR Master mix solution, 1 µL of each primer, 6 µL of template DNA, and sterile distilled water to a total volume of 20 µL. Initial denaturation (94 °C for 10 min), followed by 36 amplification cycles. Each cycle consisted of 94 °C for 30 s, 52 °C for 40 s, and 72 °C for 50 s, and the final extension step (72 °C for 5 min) was to stop the amplification. The PCR amplicons were electrophoresed on 1.5% agarose gel and were visualized by ethidium bromide staining (Poirel et al. 2011).

**Table 1:** Sequences of primers utilized in this study for PCR assessments for detection of carbapenemases genes among UPEC nosocomial isolates

Gene	Primers sequence (5′—3′)	Amplicon size (bp)	References
KPC-F	CTTGCTGCCGCTGTGCTG	489	(Tenover et al. 2006)
KPC-R	GCAGGTTCCGGTTTTGTCTC	489	(Tenover et al. 2006)
IMP-F	GGAATAGAGTGGCTTAAYTC	232	(Poirel et al. 2011)
IMP-R	TCGGTTTAAYAAAACAACCACC	232	(Poirel et al. 2011)
VIM-F	GATGGTGTTTGGTCGCATA	390	(Poirel et al. 2011)
VIM-R	CGAATGCGCAGCACCAG	390	(Poirel et al. 2011)
OXA48 F	GCGTGGTTAAGGATGAACAC	438	(Poirel et al. 2011)
OXA48 R	CATCAAGTTCAACCCAACCG	438	(Poirel et al. 2011)

### Statistical analysis

The Statistical Package for the Social Sciences (SPSS), version 22 (SPSS Inc., Chicago, IL, USA) was utilized for data coding, validation, and analysis. The data were expressed as frequencies and percentages. The Student’s *t*-test was used to contrast numeric data, whereas the Chi-square test was utilized to analyze categorical data. A binary logistic regression analysis with antecedent 95% confidence intervals (CIs) was utilized to identify relevant risk factors.

## Results

Throughout the study period, all 130 non-duplicate nosocomial strains of UPEC were isolated from 75 females and 55 males. The majority of isolates (*n* = 72, 55.4%) were obtained from ICU patients. The ages of the patients ranged from 13 to 66 years, with a median of 40 years and a mean of 40.36.

Among all UPEC nosocomial isolates, 76 (58.5%) isolates were biofilm producers. Out of which, 17 isolates (22.4%) were moderate biofilm producers and 8 (10.5%) were strong biofilm producers, while the rest (*n* = 51, 67.1%) were weak biofilm producers.

Table 2 displays infected patients’ demographic and clinical features with a biofilm- and non-biofilm-producing UPEC isolates. There were no statistically significant differences between the two groups in terms of sex, age, and the majority of comorbidities. However, malignancy, diabetes mellitus (DM), duration of hospital stay, intensive care unit (ICU) admission, prior antibiotic therapy, urinary catheterization, carbapenems resistance (*p* ≤ 0.001 for each), and resistance patterns (*p* = 0.012) were statistically significant risk factors correlated with infections by biofilm-producing isolates utilizing univariate analysis.

**Table 2:** The demographic and clinical features of cases infected by biofilm-producing and non-biofilm-producing UPEC nosocomial samples (*n* = 130)

Characteristic	Biofilm producers *N* = 76 (58%)	Non-biofilm producers *N* = 54 (42%)	*p* value
Age (M ± SD)	38.91 ± 11.31	41.76 ± 4.61	0.152
Duration of hospital stay			
≥ 7 days	64 (84.2%)	17 (31.5%)	< 0.001*
< 7 days	12 (15.8%)	37 (68.5%)	
Gender			
Female	45 (59.2%)	30 (55.6%)	0.678
Male	31 (40.8%)	24 (44.4%)	
Comorbidities			
DM	27 (35.5%)	3 (5.6%)	< 0.001*
HTN	19 (25%)	12 (22.2%)	0.714
Liver failure	7 (9.2%)	2 (3.7%)	0.223
Renal failure	11 (14.5%)	6 (11.1%)	0.575
Head trauma	6 (7.9%)	3 (5.6%)	0.605
Polytrauma	3 (3.9%)	0 (0%)	0.140
Malignancy	40 (52.6%)	13 (24.1%)	0.001*
ICU admission	57 (75%)	15 (27.8%)	< 0.001*
Prior antibiotic therapy	64 (84.2%)	17 (31.5%)	< 0.001*
Urinary catheterization	61 (80.3%)	19 (35.2%)	< 0.001*
Carbapenem resistance	17 (22.4%)	0 (0%)	< 0.001*
Resistance pattern			
MDR	23 (32.4%)	22 (44%)	0.012*
XDR	37 (52.1%)	28 (56%)	
PDR	11 (15.5%)	0 (0%)	
Carbapenemase genes			
* bla*VIM	1 (6.3%)	0 (0%)	0.797
* bla*OXA48	6 (37.5%)	0 (0%)	0.446

*M* mean, *SD* standard deviation, *DM* diabetes mellitus, *HTP* hypertension, *ICU* intensive care unit, *MDR* multiple drug resistant, *XDR* extensively drug-resistant, *PDR* pan drug-resistant, *Significant *p* value

**Fig. 2:**
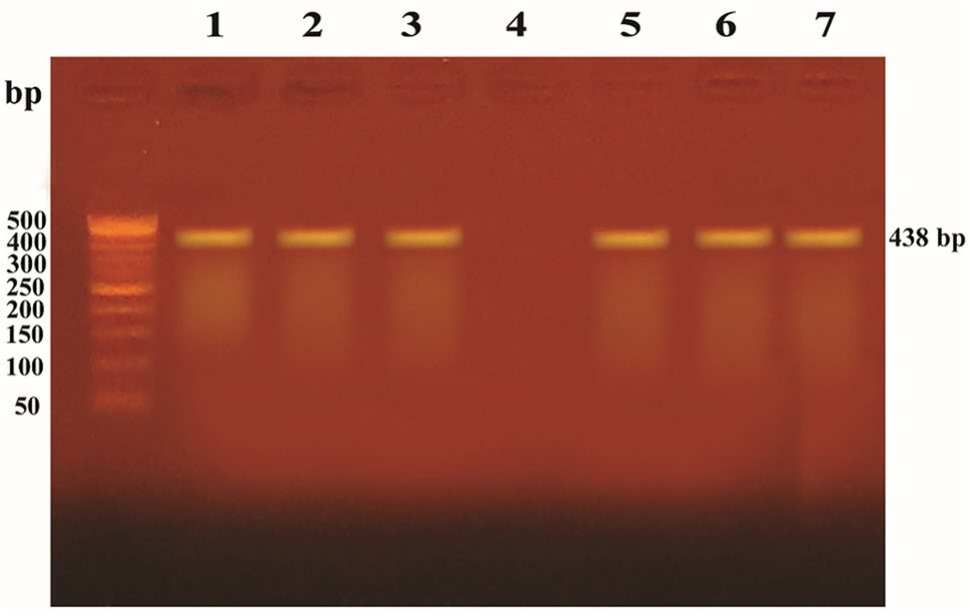
Agarose gel electrophoresis used for separation of the amplicons produced by multiplex PCR reaction for *bla*_KPC_ (498 bp) and *bla*_OXA-48_ (438bp) genes. The ladder used is 50 bp ladder, *bla*_OXA-48_ amplicon (438 bp) was detected in lanes 1, 2, 3, 5, 6, and 7 while *bla*_KPC_ amplicon (498 bp) was not detected in any lane

**Fig. 3:**
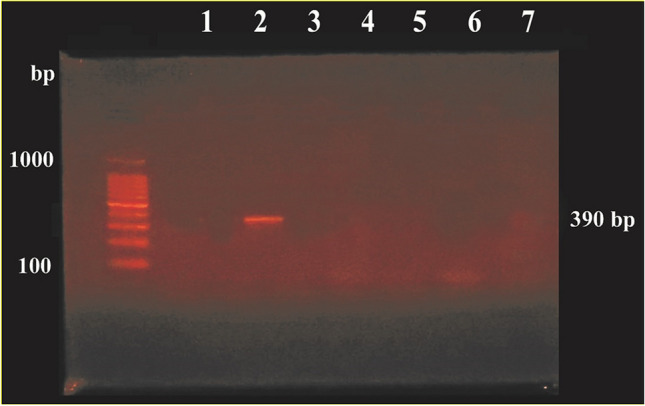
Agarose gel electrophoresis (1.5% w/v) used for separation of the amplicons produced by multiplex PCR reaction for *bla*_IMP_ (232 bp) and *bla*_VIM_ (390bp) genes. The ladder used is 100 bp ladder, *bla*_VIM_ amplicon (390bp) was detected in lane 2 while *bla*_IMP_ amplicon (232 bp) was not detected in any lane

It is noteworthy that carbapenem resistance was detected in 17 UPEC nosocomial isolates (All were biofilm producers). Six isolates harbored *bla*_OXA48_ (42.9%), and only one isolate (9.1%) was positive for *bla*_VIM_ (Figs. 2 and 3). Moreover, among the biofilm-producing isolates, 23 isolates (32.4%) were MDR, 37 isolates (52.1%) were extensively drug-resistant (XDR), and 11 isolates (15.5%) were pan-drug resistant (PDR).

Table 3 illustrates that after regression, prior antibiotic therapy was the most significant risk factor for biofilm production by UPEC (aOR = 5.782, *p* = 0.000). DM was another significant risk factor for biofilm production by UPEC (aOR = 11.222, *p* = 0.016).

**Table 3:** Logistic regression analysis for the correlation of demographic and clinical characteristics with biofilm production among the studied patients (*n* = 130)

Variables	95% CI	aOR	*p* value
Malignancy	0.048–2.443	0.343	0.285
DM	1.577–79.872	11.222	0.016*
Catheterization	0.418–6.775	1.682	0.464
ICU admission	0.834–35.591	5.447	0.077
Duration of hospital stay	0.680–1.179	0.895	0.431
Prior antibiotic therapy	4.306–57.834	15.782	0.000*
Carbapenem resistance	0	0	0.998
Resistance pattern	0.252–2.397	0.778	0.661

*DM* diabetes mellitus, *ICU* intensive care unit, *Significant *p* value

Among all UPEC nosocomial isolates, resistance rates to different antimicrobial classes were generally high and worrisome, with the highest rates reported to cefoxitin (100%) followed by cefepime, respectively aztreonam (94.6% for each). The antibiotics with the lowest resistance rates were doxycycline and tigecycline (10% for each), followed by gentamycin (18.5%). Moreover, 45 (37%) isolates were MDR, 65 (53.7%) isolates were XRD, and 11 (9.1%) isolates were PDR.

**Fig. 4:**
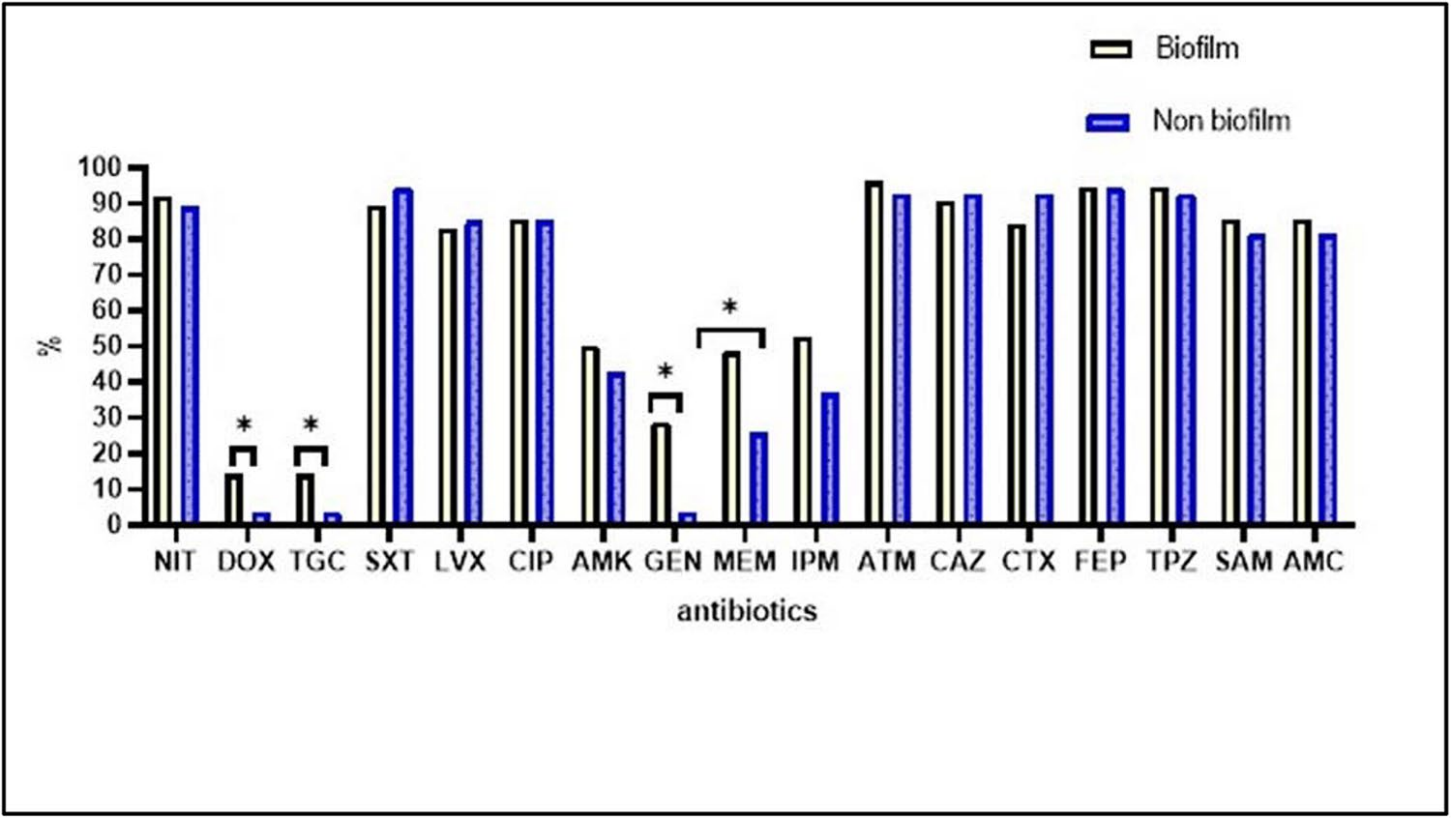
Resistance patterns of biofilm-producing and non-biofilm-producing UPEC nosocomial isolates to different antimicrobials. *Significant *p* value

Figure 4 and Table 4 show that biofilm generation in UPEC was significantly correlated with resistance to gentamycin (*p* < 0.001), meropenem (*p* = 0.009), doxycycline, and tigecycline (*p* = 0.044 for each). There was no statistically significant correlation between biofilm generation and resistance to the other antimicrobials. However, after adjusting confounding factors using logistic regression analysis, the biofilm formation was only significantly linked with resistance to gentamycin (aOR = 9.113, *p* = 0.02) (Table 4).

**Table 4:** Logistic regression analysis for the association of antibiotic resistance rates with biofilm production among the UPEC nosocomial isolates (*n* = 130)

Antibiotics	95% Confidence interval	aOR	*p* value
Doxycycline	0.123–6.981	0.942	0.942
Tigecyclin	0.934–20.73	4.400	0.061
Gentamycin	1.37–60.5	9.113	0.02*
Meropenem	0.531–3.08	1.280	0.583

*Significant *p* value

## Discussion

*E. coli* is the main etiology for UTIs, accounting for approximately 80% of community-acquired and 50% of hospital-acquired infections worldwide. In developing countries, the epidemiologic prevalence of UPEC infections ranges from 50 to 80%, compared with a rate of 3–40% in developed countries (Kot 2019). The burden of biofilm and carbapenemase production among UPEC is considered a global health crisis (Katongole et al. 2020). Furthermore, treatment of UTIs caused by UPEC, especially hospital-acquired ones, has become more challenging due to increasing resistance rates for standard antibiotics. This increase of antibiotic resistances and the emergence of MDR-UPEC are continuously associated with higher rates of inadequate empirical therapy (Kot 2019). In addition, hospitals-acquired UTIs caused by MDR-UPEC result in increased morbidity and mortality compared to those caused by susceptible strains (Flores-Mireles et al. 2015). Carbapenems have been considered the last treatment option for infections caused by MDR-UPEC. Nonetheless, the emergence of carbapenems resistance among UPEC isolates is a major concern due to their high virulence (Nasrollahian et al. 2022). Biofilm-producing UPEC is commonly associated with recurrent and complicated UTIs (Katongole et al. 2020). Due to biofilm's significance in the UPEC pathogenesis, identifying biofilm-producing strains is essential to select the most effective treatment options for these resistant strains and prevent their spread in healthcare systems.

In our study, 58.5% of all UPECs were found to produce biofilms. Recent meta-analysis research indicates that UPEC biofilm production varies globally between 13.3 and 99%. Prior epidemiological research has demonstrated that biofilm development is the most important virulence factor promoting the endemicity and chronicity of *E. coli* isolates in both nosocomial infections and hospital settings (Karigoudar et al. 2019). Notably, 84.2% of the biofilm-producing strains in our study were hospitalized for ≥ 7 days. Moreover, 75% of the isolates were obtained from ICU patients. This result would further clarify the application of biofilm-producing *E. coli* in nosocomial infections and outbreaks among high-risk ICU cases (Hu et al. 2015; Raya et al. 2019).

We reported that 52.6% of patients infected with biofilm-producing UPEC had malignancy as comorbidity. Chemotherapy and radiotherapy are commonly responsible for immunosuppression in cancer patients. Consequently, they are commonly placed on low doses of antimicrobials, which could lead to multidrug resistance. Some are catheterized, which can aid the formation of biofilms, with Rizzato et al. (2019) reporting a similar association.

Regarding urinary catheterization, 80.3% of the biofilm-producing isolates were collected from catheterized patients. Several studies agreed with our results (Zagaglia et al. 2022; Karigoudar et al. 2019). Biofilm production is the first step to catheter-associated UTI pathogenesis. Bacteria in biofilm move via the catheter into the bladder within 1 to 3 days and induce an infection (Gunardi et al. 2021; Pelling et al. 2019).

Logistic regression analysis for our data revealed that prior antibiotic therapy and DM were significant risk factors for UTIs caused by biofilm-producing isolates. Many previous studies showed that antimicrobial therapy selects antimicrobial-resistant strains commonly associated with biofilm production, providing a tough polymeric matrix impeding antimicrobial penetration (Sharma et al. 2019; Singh et al. 2017). Previous studies also indicated the relation between biofilm production and DM, as the main feature of a diabetic wound is the polymicrobial composite that modulates bacterial virulence. These microorganisms intercommunicate and build a sophisticated polymicrobial biofilm population (Zagaglia et al. 2022; Raya et al. 2019; Pouget et al. 2020; Afonso et al. 2021). Diabetes is a common risk factor for recurrent UTIs. In addition to diabetes-associated impaired host defense mechanisms resulting in an increased risk of infections, diabetic neuropathy affects the ability of the bladder to sense the presence of urine in the bladder, allowing urine stasis that favors UTIs (Nitzan et al. 2015). Moreover, the higher glucose level in the urine is known to improve the growth of bacteria in the urine and increase the probability of infection. In our study, the increased risk of UTIs caused by biofilm-producing isolates in diabetic patients could be attributed to the previously reported data that biofilm production phenotype is modulated in response to a variety of environmental signals, including available glucose levels. Additionally, quorum signaling compounds such as insulin regulate the processing of the environmental signals that regulate biofilm phenotype (Sewify et al. 2016; Raya et al. 2019; Patel et al. 2021).

In this study, 22.4% of biofilm-producing isolates were found to be carbapenem-resistant. In intensive care units, carbapenem resistance in Gram-negative bacteria, such as UPEC, is an emerging global problem. The World Health Organization (WHO) added carbapenem-resistant organisms (CRO) to its list of priority pathogens in 2017 due to their alarming spread, which could severely limit future therapeutic treatment options (Tacconelli et al. 2018). Several mechanisms contribute to carbapenem resistance, including the production of carbapenemases or a combination of porin deficiency and other β-lactamases (Karigoudar et al. 2019). Among our carbapenem-resistant isolates, six (42.9%) isolates harbored *bla*_OXA48_, and only one (9.1%) isolate was positive for *bla*_VIM_. In a study by Gurung et al. (2020), *bla*_OXA48_ was detected in 33.3% among UPEC while Bindayna et al. (2022) reported *bla*_VIM_ in 8.5% of *E. coli* isolates. Gatya Al-Mayahie et al. (2022) reported that *bla*_OXA-48_ was the most frequent variant (57.8%), while *bla*_VIM_ was detected in 10.5%. In our study, none of the isolates were positive for *bla*_KPC_ and *bla*_IMP_ genes, which agreed with Loqman et al. (2021).

All biofilm-producing isolates in this study displayed resistance patterns of MDR, XDR, and PDR. However, biofilm-producing organisms were more resistant to gentamycin, meropenem, doxycycline, and tigecycline than other antibiotics. Nevertheless, logistic regression analysis showed that biofilm formation was significantly linked with resistance to gentamycin (aOR = 9.113, *p* = 0.02), indicating a potential association between the biofilm genes and aminoglycoside resistance genes. Our findings agree with previous studies that recommend biofilms to be correlated with elevated resistance to antibiotics (Katongole et al. 2020; Singh et al. 2017).

This study has some limitations. First, the study included patients from a single center in a particular geographic region, so the findings cannot be generalized to the whole population. In addition, this study included small number of isolates and it did not investigate the biofilm-inducing genes or other carbapenem-related genes other than *bla*_KPC_, *bla*_IMP_, *bla*_VIM_, and *bla*_OXA-48_ genes at the molecular level. Further molecular-based epidemiologic multi-center studies from different regions are required. Findings from these studies can contribute to controlling the spread of highly virulent UPEC and strengthen infection prevention strategies.

## Conclusions

In this study, the high prevalence of UPEC nosocomial isolates that produce bio-film is alarming and concerning. The escalating rate of antibiotic resistance is a significant cause for concern. Our findings highlight the importance of identifying potential risk factors associated with biofilm-producing UPEC nosocomial infection. These findings may aid health authorities and decision-makers in regulating the continuous selection and spread of this dangerous trait within hospitals and improving the quality of care for patients.

## Data Availability

No datasets were generated or analyzed during the current study.
